# The gesture ‘Touch’: Does meaning-making develop in chimpanzees’ use of a very flexible gesture?

**DOI:** 10.1007/s10071-017-1136-0

**Published:** 2017-10-24

**Authors:** Kim A. Bard, Vanessa Maguire-Herring, Masaki Tomonaga, Tetsuro Matsuzawa

**Affiliations:** 1 Psychology Department, University of Portsmouth, King Henry I Street, Portsmouth, PO1 2DY UK; 2 Primate Research Institute, Kyoto University, Inuyama, 484-8506 Japan; 3 Institute for Advanced Study, Kyoto University, Kyoto, 606-8501 Japan; 4 Monkey Centre, Inuyama, 484-0081 Japan

**Keywords:** Great apes, Infants, Development, Non-verbal communication

## Abstract

In this bottom-up study of gesture, we focused on the details of a single gesture, Touch. We compared characteristics of use by three young chimpanzees with those of 11 adults, their interactive partners, housed in a semi-natural social group at the Kyoto University Primate Research Institute (KUPRI) in Japan. Five hundred eighty-one observations of the gesture Touch were collected across a four-year time span. This single gesture had 36 different forms, was directed to 70 different target locations on the body of social partners, and occurred in 26 different contexts. Significant differences were found between infant and adult initiators in the form, target locations, and contexts of the gesture Touch. There was a wide diversity in form–location patterns within each context, and there were no form–location patterns specific to particular contexts. Thus, we demonstrate that this gesture exhibits flexibility in form and flexibility in use. The results from this study illustrate the importance of contextualized meaning in understanding flexibility in the gesture use of great apes.

**Electronic supplementary material:**

The online version of this article (doi:10.1007/s10071-017-1136-0) contains supplementary material, which is available to authorized users.

## Introduction

Primates have long been used as models for trying to understand the developmental processes that underlie human communication, given humans close genetic relationship with chimpanzees. Studies of ape gestures have tended to focus on top-down demonstrations of underlying cognitive processes (e.g. intentionality: Bard 1992; Tomasello et al. 1985), establish repertoires of intentionally communicative gestures (e.g. Cartmill and Byrne 2010; Frohlich et al. 2016; Genty et al. 2009; Goodall 1968; Graham et al. 2016; Hobaiter and Byrne 2011; Liebal et al. 2006; Pika et al. 2003, 2005; Roberts et al. 2012; Savage-Rumbaugh et al. 1977), or focus on the evolution of the cognition underlying communication or language (e.g. Arbib et al. 2008). Few have focused on the details of the form of the gesture to illuminate communicative behaviour. Here we apply a different process of developing a gestural repertoire, one that relies on bottom-up processes, to fully describe a gesture used by chimpanzees, the gesture Touch. Among the variety of gestures found in the repertoire of apes, our preliminary evidence suggested that this one was among the most flexibly used and flexibly displayed (Herring 2013), and so we choose to investigate only this gesture. We specify the form used, in terms of the signaller’s behaviour, and the possible sensory perceptions of the receiver, in terms of the places on the body that are touched, i.e. the target locations. In line with our ideas that gestures develop (e.g. Bard et al. 2014b), we compare the use of this gesture in infants and adults. With this detailed picture of a single gesture, we evaluate the extent to which its form and use vary across contexts, individuals, and age categories (i.e. whether Touch is rigid or flexible, a la Tomasello et al. 1985).

It has been proposed that the variability in gesture use is due to the fact that gestures are used to convey non-urgent communicative content, and therefore can be used more flexibly (Tomasello and Zuberbuehler 2002). In contrast, vocal communication is thought to primarily convey evolutionary urgent information (predator detection, food sources, etc.) and therefore shows little flexibility (contra Lameira et al. 2016 for orangutans; Perlman and Clark 2015 for gorillas; Hopkins and Savage-Rumbaugh 1991 for bonobos). Therefore, gestural communication may provide important information into the evolution of communication, due to the learning-based and flexible nature of gestures (e.g. Pika et al. 2005; Pollick and de Waal 2007).

One of the assumptions inherent in the study of gestures in apes is that gestural development proceeds from mechanically effective actions to abbreviated acts that only serve a communicative purpose, in a process Tomasello and colleagues have called ontogenetic ritualization (based on the ethological concept of phylogenetic ritualization, as discussed by Perlman et al. 2012). This process is thought to account for the presence of idiosyncratic gestures, as ontogenetic ritualization occurs between a specific signaller and specific social partner (e.g. in a mother–infant dyad: Tomasello et al. 1985). Other learning processes have been suggested for the development of gestures, including genetic channelling with repertoire tuning (Hobaiter and Byrne 2011), intersubjective co-construction (Bard et al. 2014b), social negotiation (Frohlich et al. 2016), and non-ritualization types of social learning (Marentette and Nicoladis 2012; Perlman et al. 2012). It is an empirical question whether there is flexibility in the structure of gestures, such that a gesture can have different forms while retaining the same communicative message. Only a few studies have described the morphology of gestures, but those analyses were fruitful in determining a lack of morphological support for a core concept of the ontogenetic ritualization theory (Bard et al. 2014b; Hobaiter and Byrne 2011; Perlman et al. 2012), and instead support the graded nature of gestural communication (e.g. Roberts et al. 2012). In general, exploring this type of flexibility in the structure of gestures is under investigated.

Flexibility in the function of gestures can be demonstrated in a wide variety of ways. Flexibility can be shown when a single gesture is used across multiple contexts, or multiple gestures are used for the same communicative purpose within a single context. Flexibility can be shown by individuals, or by differences in gesture use across groups. Flexibility can be shown when the gesturer exhibits control over when the signal is produced, for instance, using a visual gesture only when the partner is visually attending, or tactically deploying gestures from different modalities based on the attentiveness of the recipient. Flexibility can be shown in the capacity to develop new elements in a gestural repertoire.

Chimpanzees display a flexible use of gestures in all these ways. Chimpanzees are sensitive to the attentional status of the social partner who is the intended recipient of their gestures. At a core level, gestures are only emitted in the presence of a communicative audience (Leavens et al. 1996). In most instances, a visual gesture is used if the recipient is already attending visually (Hostetter et al. 2001; Tomasello et al. 1994, 1997). If the intended recipient is not visually attentive, then chimpanzees can use a tactile or auditory gesture instead (Leavens et al. 2004a, b). Another way that chimpanzees ensure their visual gestures are received is to change their spatial location relative to their audience, moving themselves into the visual field of their intended recipient, before using a visual gesture (Liebal et al. 2004b). It is clear that chimpanzees show flexibility in the sense that they tailor the modality of gesture use to conform with the attentional status of recipients.

The issue of the meaningfulness of gestures has been tackled directly by ascertaining communicative goals. The flexibility of gestures in wild chimpanzees is demonstrated since there were, on average, between 4 and 5 satisfactory outcomes for each gesture, with only 10 of the 66 gestures having a single satisfactory outcome (Hobaiter and Byrne 2014). Interestingly, in captive orangutans most gestures were tightly associated primarily with a single intentional meaning (Cartmill and Byrne 2010). It is not clear, however, comparing these two studies, whether the different degree of flexibility in meaning relates to differences between species or between settings (Leavens et al. 2017). We reasoned that perhaps knowing the form of gestures will aid in the determination of meaning (e.g. Hobaiter and Byrne 2011 were able to distinguish actions from gestures by investigating differences in the gesture morphology).

Another aspect of flexibility is an ability to develop or use new gestures that are not found in species-typical repertoires. For example, captive chimpanzees use the manual gesture, point (Leavens et al. 1996; Leavens and Hopkins 1998), even though the gesture is rarely observed in wild chimpanzees (but see Hobaiter et al. 2013). Chimpanzees use indexical and whole-hand pointing to direct human attention to objects, such as food (Leavens et al. 1996, 2004a, b, 2005; Leavens and Hopkins 1998) or a tool needed to obtain food (Russell et al. 2005). It appears that chimpanzees develop pointing to solve the Referential Problem Space. The Referential Problem Space exists when there is a barrier between an individual and their goal object (which can be cage mesh—in the case of captive chimpanzees, or inability to locomote—in the case of young human infants), but another individual, with whom there is a communicative history, has access to the goal object (see Fig. 2: Leavens et al. 2008). Pointing solves the referential problem, by indicating the desired goal object to the communicative partner. Pointing develops in this context in young infants, and captive chimpanzees (Leavens et al. 2005). This concept of the Referential Problem Space is helpful in directing researchers’ attention to the types of situations in which one would expect pointing to occur, and explains the few instances of pointing observed in wild chimpanzees, as well (Hobaiter et al. 2013). These sets of results demonstrate that chimpanzees have flexibility in gesture use in the sense of developing new gestures (Leavens et al. 2005; Liebal et al. 2004a).

The most widely used definitions of flexibility involve multiple uses (i.e. contexts) for single gestures, or a single gesture used in multiple contexts (Tomasello et al. 1985, 1989, 1994, 1997; Maestripieri 1996; Maguire-Herring and Bard, in prep). Rigidity is found when a gesture is specific to a single context. Interestingly, when making group comparisons, in some instances one group may use a gesture flexibly, while another group may use the same gesture rigidly. For example, the gesture ‘holding hand out’ was observed in a group of wild chimpanzees in Eastern Africa only in the context of food begging, i.e. rigidly (observed by Kortland, described in Reynolds 1967), but a captive group of chimpanzees at the Arnhem Zoo used this gesture in the contexts of food begging, body contact, support during conflict, and formation of alliances, i.e. flexibly (de Waal 1982). Many previous studies have shown that the meaning of a gesture changes with context in chimpanzees (Hobaiter and Byrne 2014; Roberts et al. 2012; Tomasello et al. 1989, 1994, 1997).

It can be difficult to make comparisons of gestures across studies due to differences in criteria for which behaviours constitute a gesture and the operational definitions for particular gestures. Sometimes gestures are classified solely by their function, i.e. communicative meaning. For example, the ‘submissive behaviours’ are defined by de Waal (1982) as kissing, directed to the neck, feet, or chest of the dominant animal, whereas Goodall (1986) describes submissive behaviour as touching the lips, thighs, top of head, genitals or shoulder of the dominant chimpanzee. Although the Gombe chimpanzees do kiss, they apparently do not kiss in a submissive context (Goodall 1986). Other times gestures are classified by their general visual appearance, e.g. reach. We suggest that it is important to classify not only the form of the gesture, but also to classify more precisely where it is directed. Although ‘reach’ may occur across many contexts, for example, it is possible that ‘reach to the mouth’ may be found in more limited contexts, e.g. submissive contexts. For example, when infant chimpanzees use a gesture to initiate grooming they direct it to a specific spot that will be groomed (e.g. by looking first, then touching the spot: Bard et al. 2014b; by directed scratches: Pika and Mitani 2006). Here we include the location on the body of the recipient where each Touch landed, as this might impart meaning for the recipient (i.e. based on the direct sensory perception of the Touch).

As an initial investigation, we present here a detailed study of the form, target location, and context for a single gesture Touch. Touch has been included as a specific gesture in some previous studies of chimpanzees. For example, Call and Tomasello (2007), Liebal et al. (2004a), and Schneider et al. (2012) use Gentle touch. Schneider et al. added two other forms of Touch, Lip–lip touch, and Touch with genitals. In their sample of eight chimpanzees less than 20 months of age, however, they found that only Lip–lip touch occurred, and only in one infant chimpanzee. Others limit aspects of Touch, for example, Tomasello et al. (1985, 1994) limit Touch to a single target location of Side, whereas Hobaiter and Byrne (2011) limit Touch to particular forms, i.e. palm and or fingers. Interestingly, Gentle touch was very frequently used one in a sequence of gestures, across a variety of contexts, by a group of 19 mixed age captive chimpanzees (Liebal et al. 2004a), and Touch was used in two field settings, by most mothers and most infants, to initiate carrying in the context of joint travel (Frohlich et al. 2016). One benefit of a singular focus on just one gesture is that we can present a level of detail that might be difficult to convey with multiple foci. Another benefit is that we can evaluate whether there are any consistencies in the form of the gesture when used across different contexts, or when used by infants versus adults. Of course, a limitation is that conclusions can only be made, as a result, on the sole gesture of Touch.

A larger study documented 36 different gestures exhibited by the current chimpanzees (Table S1 from Herring 2013), and in this paper, we investigate the flexibility of a single gesture, Touch. We suggest that by specifying both the forms and the target locations of the gesture we will increase our understanding of flexibility in form and use, and thereby illuminate the process of meaning-making in gesture. We investigate whether there were patterns in the combination of form and target location that disambiguate meaning. Finally, we investigated the extent to which there were differences between adult and infant chimpanzees that would support claims of learning in the use of this gesture.

## Methods

### Subjects

**Fig. 1:**
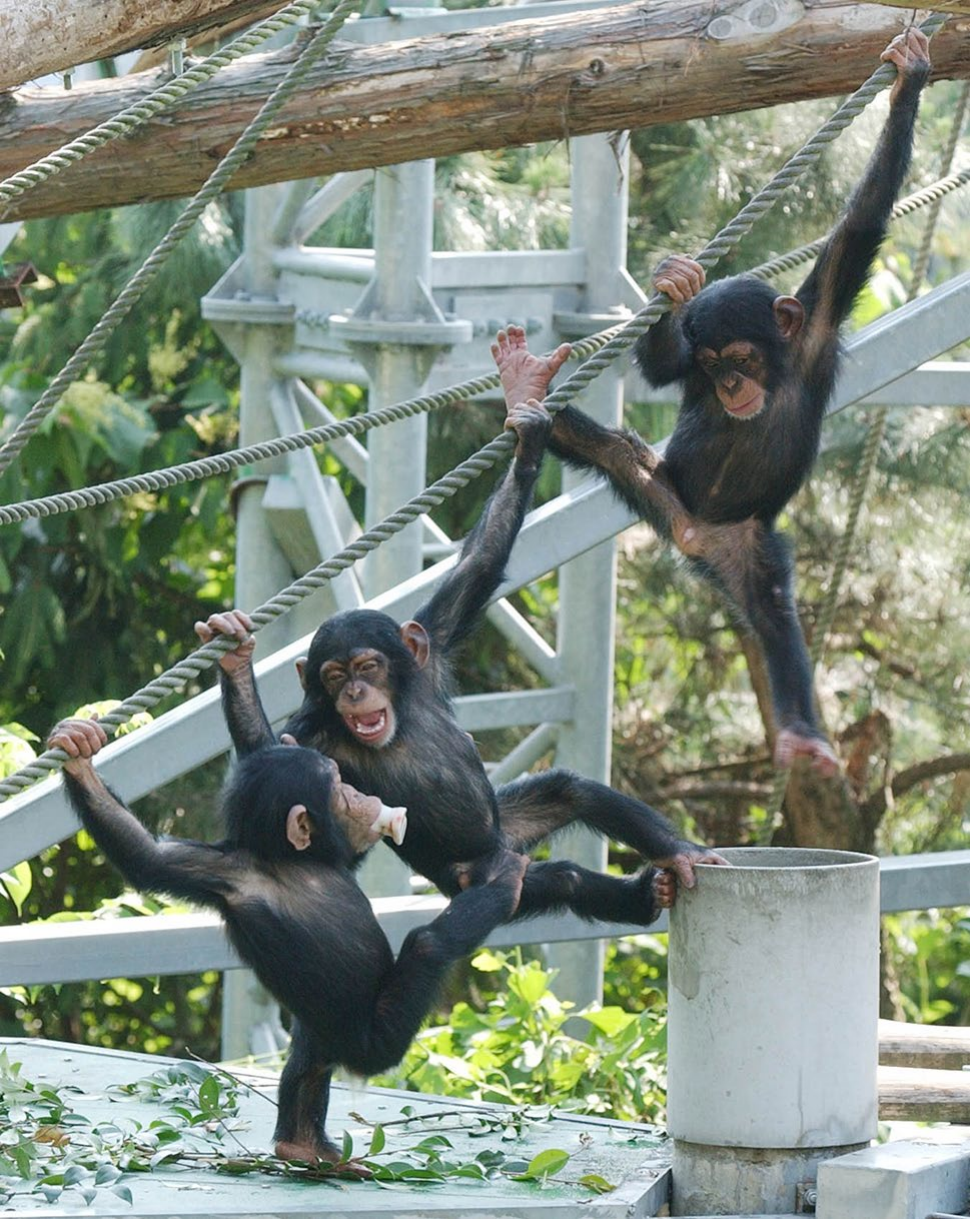
Three focal chimpanzees at the Primate Research Institute, Kyoto University, Japan. *Photo credit*: Akihiro Hirata

The subjects for this study were three young chimpanzees (two females and one male: Fig. 1) and the 11 adult chimpanzees (eight females and three males) with whom they interacted (Table 1). All subjects were housed in a semi-natural social group at the Kyoto University Primate Research Institute (KUPRI), Kyoto, Japan (see: http://langint.pri.kyoto-u.ac.jp/ai/ for further information: Matsuzawa 2003; Matsuzawa et al. 2006).

**Table 1:** Subject information

Sex	Name	Date of birth	GAIN ID	Additional information
M	Ayumu	April 2000	0608	Infant
F	Cleo	June 2000	0609	Infant
F	Pal	Aug. 2000	0611	Infant
F	Puchi	1966	0436	Adult
M	Gon	1966	0437	Adult
F	Reiko	Dec. 1966	0432	Adult
F	Mari	June 1976	0274	Adult
M	Akira	June 1976	0435	Adult Ayumu and Pal’s father
F	Ai	Oct. 1976	0434	Adult Ayumu’s mother
F	Pendesa	Feb. 1977	0095	Adult
F	Chloe	Dec. 1980	0441	Adult Cleo’s mother
F	Popo	March 1982	0438	Adult
M	Reo	May 1982	0439	Adult Cleo’s father
F	Pan	Dec. 1983	0440	Adult Pal’s mother

**Fig. 2:**
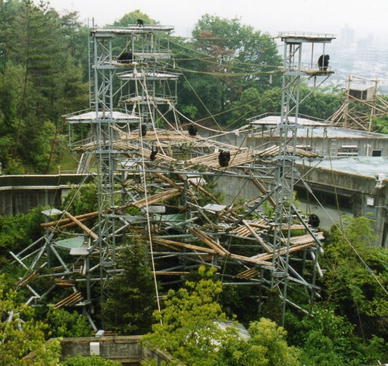
Outdoor facility at the Primate Research Institute, Kyoto University, Japan. *Photo credit*: Tetsuro Matsuzawa

The chimpanzees enclosure was 700 metres square and semi-natural, with walkways giving access to indoor and three outdoor enclosures (Fig. 2). The indoor enclosures included large climbing structures, ropes, and platforms. The largest of the three outdoor enclosures consisted of a large five-story climbing structure equipped with ropes and ladders. The two smaller enclosures were similar in design and included climbing structures and platforms. All three outdoor enclosures consisted of over 500 natural trees, comprised of 60 different species, grass, larger climbing structures, ropes, running water, and platforms. The chimpanzees were able to remain outdoors all day but also freely travelled into the indoor enclosures and into indoor experimental booths to participate in studies and interact with their human caretakers as well (Matsuzawa 2003, 2007).

### Coding procedures

A total of 78 h of focal infant videotaped observations were collected and scored (Ayumu 27 h, Cleo 24 h and Pal 27 h) from when the infants were between 15 and 60 months old. Each video was one hour long and followed one focal individual (one of the infants). Gestures were coded using an event coding and cross-classifying events scheme (Bakeman and Quera 2011). Each time the gesture Touch was observed directed to an infant, or used by an infant, the video was stopped, the section re-watched and the following events were coded: (1) the initiator and the receiver of the gesture; (2) the context in which the gesture occurred; and (3) a complete written description of what the gesture looked like (i.e. the form of the hand) and how it was directed to the social partner (target location).

We defined a gesture as a voluntary movement of the limb(s) or body, directed towards another individual that appeared to serve a communicative function. The gesture Touch was defined as an individual voluntarily using a body part to make tactile contact with another individual for a communicative purpose. Note that Touch was reserved for a gesture that was distinct from the other 15 tactile gesture types (see Table S1 for list of 36 gestures: Herring 2013).

### Analyses

Initial summaries included all forms, all target locations, and all the contexts in which the gesture Touch was found to occur. Chi-square tests were used to determine if infants and adults differed, if there was an overall relation between form and context, if there was a relation between target location and context, and whether there were form–locations patterns found within contexts or across initiator. In order to meet the assumptions of the Chi-square analyses (i.e. to minimize the number of cells with low expected frequencies), some of the categories were collapsed. Both the complete lists and the reduced lists of categories are given in the results. Adjusted residuals were calculated ((observed# − expected#)^2^/expected#) for each cell and are reported when there was a significant Chi-square (note the Chi-square statistic is the sum of the [absolute values] adjusted residuals for all cells in the matrix). Adjusted residuals can be used to identify those cells that contribute significantly.

### Reliability

The entire corpus was coded, and 36 different gestures types (including Touch) were documented (Table S1: Herring 2013). An inter-observer reliability assessment was conducted on this coding scheme. A naive observer was trained, and a randomly selected 20% of the corpus was rescored, in 10-min segments. The inter-observer reliability assessment verified that an independent observer recorded the same information as the primary coder. All categories (initiator, gesture type, and context) were coded reliably. i.e. at a level of 90% or higher. Inter-observer reliability was 92% for Ayumu, 95% for Cleo and 95% for Pal.

A single person (VH) was the primary coder for the entire corpus, and to determine whether there was any drift in scoring from the beginning to the end, intra-observer reliability assessment was conducted. The results were similar, above 90% agreement for initiator, gesture type, context, and by subject (i.e. 91% for Ayumu, 96% for Cleo, and 97% for Pal).

## Results

There were 581 instances of the gesture Touch. Here we present the original and revised (reduced) lists of the form of the gesture, the target location, and the context, with Chi-square analyses to ascertain whether these aspects varied with the age category of the initiator (i.e. infant versus adult chimpanzees). Within each context, we also investigate whether there are specific forms associated with specific target locations.

### Form

There were 36 different specifications of the form of the gesture Touch, some of which occurred frequently and some very rarely (Table 2). Two forms occurred frequently (10% or more) for infants (Hand and Fingers), and four forms occurred frequently for the adults (Hand, Holds with hand, Fingers, and Finger tips).

**Table 2:** Observed forms of the gesture Touch (raw frequency [#] and percentage [%]) for infant and adult initiators

Form	Infant initiator	Adult initiator
	# (%)	# (%)
**Knuckles**	13 (5%)	21 (7%)
Bent knuckles	1 (< 0.5%)	2 (1%)
**Hand (s)**	109 (40%)	108 (35%)
Open hand	1 (< 0.5%)	0 (0%)
Hand-back	7 (3%)	1 (< 0.5%)
Hand-back, bent	1 (< 0.5%)	0 (0%)
Bent hand	0 (0%)	2 (1%)
Bent hand-side	2 (1%)	0 (0%)
Palm	4 (1.5%)	3 (1%)
**Fingers**	34 (12.5%)	43 (14%)
Fingers-bent	1 (< 0.5%)	0 (0%)
Fingers-back	2 (1%)	8 (3%)
Pinky-side	0	4 (1%)
Finger-single	1 (< 0.5%)	1 (< 0.5%)
**Thumb**	1 (< 0.5%)	0 (0%)
Thumb-side	0	1 (< 0.5%)
Thumb-tip	0 (0%)	3 (1%)
**Finger tips**	20 (7%)	38 (12%)
Finger tip-index	1 (< 0.5%)	0 (0%)
**Index finger**	19 (7%)	9 (3%)
**Foot (feet)**	8 (3%)	2 (1%)
Stands on	3 (1%)	0 (0%)
Toes	0 (0%)	1(< 0.5%)
**Other**		
Arm	3 (1%)	2 (1%)
Mouth	1 (< 0.5%)	1 (< 0.5%)
Legs	1 (< 0.5%)	0 (0%)
Lips	0	1 (< 0.5%)
**Hand** **+** **action**		
Hand on	3 (1%)	3 (1%)
Hand-push	2 (1%)	4 (1%)
Hand around-squeeze	1 (< 0.5%)	0 (0%)
Hand over	1 (< 0.5%)	0 (0%)
**Holds**	4 (1.5%)	0 (0%)
With?	1 (< 0.5%)	0 (0%)
With arm	1 (< 0.5%)	0 (0%)
With foot	2 (1%)	0 (0%)
**Holds with hand**	25 (9%)	50 (16%)
**Total**	273 (100%)	308 (100%)

Bolded form categories indicate the higher-order categories. The forms that are indented were summed with the higher-order categories in later analyses

To assess whether there were differences in form as a function of initiator, or context, infrequent categories were collapsed. Infrequent variant forms that specified parts were subsumed into the larger categories (e.g. these are indicated with an indent in Table 2). Two new categories were created, Hand Action, which included touches with the hand that also included some additional action (on, over, and push), and Other, which included infrequent forms of Touch with Arm, Mouth, Legs, and Lips. This reduced the number of form categories from 36 to 10 and reduced the range of frequency from 0 to 112 (with 36 categories) to 8 to 238 (for ten categories: Table 3).

**Table 3:** Adult and infant chimpanzees use significantly different forms of the gesture Touch

Form	Adult initiator		Infant initiator		
	Frequency (%)	Adj. residual	Frequency (%)	Adj. residual	Total #
Knuckles	23 (7.5%)	1.2	14 (5%)	− 1.2	37
Fingers tips	38 (12%)	1.9	21 (8%)	− 1.9	59
Finger	60 (19.5%)	1.7	39 (14%)	− 1.7	99
Index finger*	9 (3%)	**− 2.3**	19 (7%)	**2.3**	28
Hand*	114 (37%)	**− 2.1**	124 (45%)	**2.1**	238
Hand action	7 (2%)	− 0.2	7 (3%)	0.2	14
Hold with hand*	50 (16%)	**2.5**	25 (9%)	**− 2.5**	75
Hold other**	0 (0%)	**− 3.0**	8 (3%)	**3.0**	8
Feet*	3 (1%)	**− 2.4**	11 (4%)	**2.4**	13
Other	4 (1%)	− 0.5	5 (2%)	0.5	9
Total	308 (100%)		273 (100%)		581

Asterisk indicates form categories that differed between infants and adults. Bold indicates adjusted residuals that are significant. * *p* < .05; ** *p* < .01

Adults and infants differed significantly in the forms of Touch, Chi-square (9) = 34.57, *p* < .001 (Table 3). The Hand, Index Finger, Feet, and Hold other forms were used significantly more frequently than expected by infants. Adults used Hold with the hand significantly more frequently than expected. The remaining five form categories were used equally often by adults and infants.

### Target location

The chimpanzees used 70 different target locations for the gesture Touch, when specific details were included such as laterality and very specific body parts (e.g. lips, right ear, left forearm, right index finger). There was a single instance in which Touch was targeted to an arm and a leg together. Since the arm was mentioned first (and was likely better perceived by the recipient), the target location of this Touch was recoded as Arm. The 70 different locations were reduced to the 18 body areas listed in Table 4, with frequencies ranging from 4 to 76 per category.

**Table 4:** Different target locations for the gesture Touch (with frequency and percentage[%]) in chimpanzees

Target location	Total Frequency (%)
**Arm**	68 (12%)
Shoulder	8 (1%)
Leg + arm	1 (0.2%)
**Back**	74 (13%)
**Belly**	31 (5%)
**Chest**	11 (2%)
Arm pit	5 (1%)
**Chin**	16 (3%)
**Face**	35 (6%)
**Feet**	52 (9%)
Toes	3 (0.5%)
**Anogenital**	
Genitals	4 (1%)
Bum	27 (5%)
**Hand(s)**	76 (13%)
Finger(s)	7 (1%)
**Head**	49 (8%)
**Leg**	61 (10%)
**Neck**	13 (2%)
**Side**	40 (7%)
Total	581 (99.52%)

Bolded form categories indicate the higher-order categories. The forms that are indented were summed with the higher-order categories in later analyses

Categories with low frequency were collapsed into larger body locations to conduct statistical analyses, resulting in 13 target areas (Table 5). This included categorizing Toes with Feet, Fingers with Hands, Shoulders with Arm, and Arm pit with Chest, and putting the categories of Bum and Genitals together into a new category Anogenital region.

**Table 5:** Adult and infant chimpanzees direct the gesture Touch to significantly different locations on the body of the recipient

Target location	Infant initiator		Adult initiator	
	Frequency (%)	Adj. Residual	Frequency (%)	Adj. Residual
Arm	40 (15%)	0.9	37 (12%)	− 0.9
Back*	43 (16%)	**2.1**	31 (10%)	**− 2.1**
Belly**	7 (3%)	**− 2.8**	24 (8%)	**2.8**
Anogenital	13 (5%)	− 0.6	18 (6%)	0.6
Chest	10 (4%)	1.3	6 (2%)	− 1.3
Chin*	12 (4%)	**2.3**	4 (1%)	**− 2.3**
Face	20 (7%)	1.2	15 (5%)	− 1.2
Foot**	16 (6%)	**− 2.8**	39 (13%)	**2.8**
Hand	45 (17%)	1.4	38 (12%)	− 1.4
Head	19 (7%)	− 1.2	30 (10%)	1.2
Leg	24 (9%)	− 1.3	37 (12%)	1.3
Neck	9 (3%)	1.6	4 (1%)	− 1.6
Side	15 (5%)	− 1.2	25 (8%)	1.2
Total	273 (101%)		308 (100%)	

Asterisk indicates target location categories that differed between infants and adults. Bold indicates adjusted residuals that are significant. * *p* < .05; ** *p* < .01

There was a significant association between initiator and target location, Chi-square (12) = 35.80, *p* < .001 (Table 5). In particular, infants targeted the Chin and Back of the social partner significantly more than expected, whereas adults directed their Touch gestures significantly more often than expected to the Belly and Feet of the social partner.

### Context

There were 26 different contexts recorded, including six blended contexts (Table 6). Contexts were reduced to 13 to conduct statistical analyses (Table 7). The blended contexts were collapsed: all contexts that blended with Attention getting were placed in Attention getting context, and of the remaining blended contexts, those that were blended with Greeting were placed in Greeting. The rationale was that Attention getting or the Greeting was the initial context that blended into the second context. The final blended context, Contact/Play was placed into a new category of Other affiliative, along with Appease, Genital inspection, Tandem walk, and Reassurance. Safeguard was collapsed with Retrieve, and Submissive was collapsed with Dominance. Finally, all the contexts related to food were put together. This resulted in 12 contexts, with a final one that was undefined (Table 7).

**Table 6:** All contexts in which the gesture ‘Touch’ was observed are listed. Context categories in bold indicate the reduced number of contexts used in the analyses

**Attention getting** ^q^
Attention getting—An individual uses a gesture to alert or otherwise manipulate another’s attention to gain a focus on the self
Play/Attention getting—The gesture is used to gain the partner’s attention and to initiate play
Attention getting/Contact—The gesture is used to gain the partner’s attention and to initiate contact
Greet/Attention-getting—The gesture is used to gain the partner’s attention and to greet
**Comfort** ^a,b,f,k,m,p^
Comfort—When an individual is upset, frightened, nervous (usually accompanied with vocalizations) and another individual attempts to calm the upset individual
**Contact** ^a,b,f,g,i,j,m^
Contact—An individual passively sits next to (with physical contact) or in close proximity to (within an arm’s length) another individual
**Dominance/submission** ^a,b,c,f,g,h,k,n–p^
Dominance—The dominant individual may acknowledge the subordinate’s submissive gestures, e.g. by touching. The dominant chimpanzee may aggress, grab, or slap the subordinate for no apparent reason other than to assert one’s dominance
Submissive—In this context, either a subordinate individual approaches or is approached by a dominant individual and the subordinate individual assumes the appropriate postures and gestures, such as crouching or greetings with touches or kisses
**Food beg/share/steal** ^a,b,d,e,f,j,n,o,q^
Food stealing—In this context, the recipient is in possession of food, and the focal individual gestures to distract the recipient in order to take the food
Food beg and/or Food share—In this context, an individual is in possession of food. Another individual, without food, may touch, or lip touch at the food or at the possessor. The individual with food will sometimes allow the other to take some food or will sometimes offer
**Greet** ^b,f,k,p^
Greeting—Greeting is defined as when one individual approaches another individual and briefly pauses in front of them and may exhibit a number of gestures such as a hug, touch, kiss, etc.
Play/Greet—The context includes a gesture that is used to greet the social partner and also to initiate a play bout
Greet/Contact—The context includes a gesture that is used to greet the social partner and also to initiate contact
**Groom** ^a–c,f–q^
Grooming—In this context, individuals sit in close proximity and use their hands, fingers, and lips to remove skin particles and debris from the other individual’s hair and skin
**Locomotion** ^b–f,i–k,n–q^
Locomotion—An individual moves from one part of the enclosure to another, with a minimum of two steps
**Nurse** ^c,f,j,q^
Nursing—This context is clearly limited to an adult female and her young offspring. In this situation, the offspring is attempting or actually suckling from the mother’s breast
**Play** ^a–q^
Play—Social interactions that can involve tickling, chasing, and wrestling. Frequently during these activities, one or both partners exhibit a ‘play face’ and may pant grunt
**Retrieve/safeguard** ^a,f^
Retrieve—One individual extends assistance or tries to change the location of the other. In either case, the partner moves into proximity or ventral contact with the actor
Safeguarding—Safeguarding involves acting to protect or to prevent harm occurring to another
**Other affiliative**
Appeasement^a,b,f,k,q,r^– The context of comforting another who is distressed, e.g. by the aggression of the focal or by a third party
Genital inspection—This context is distinct from grooming and relates to when the chimpanzees are engaged with a specific focus on exploring or inspecting the anogenital region
Reassurance^a,b,f,k^—Similar to the context of comfort, in a reassurance context, normally the individual is not visibly or vocally distressed. The goal appears to be indicating that everything is alright, rather than calming the social partner, *per se*
Tandem walk^d–g,q^—Either one or both individuals will place their arms around or hold on to the other’s back and walk side by side together
Play/Contact—In some cases it is unclear whether the gesture is used to initiate play or just to make contact with another individual
**Undefined**
Undefined—This category was used when the context was unclear or could not be determined, even after repeated viewings

Contexts that have been similarly described by other gesture researchers are indicated by the following superscripts

^a^Bard (1994), Bard et al. (2014b)

^b^Berdecio and Nash (1981)

^c^Call and Tomasello (2007)

^d^Frohlich et al. (2016)

^e^Frohlich et al. (2017)

^f^Goodall (1968, 1986)

^g^Hobaiter and Byrne (2011)

^h^Liebal et al. (2004a)

^i^Nakamura and Sakai (2014)

^j^Nicolson (1977)

^k^Nishida (1970)

^l^McCarthy et al. (2013)

^m^Plooij (1978)

^n^Roberts et al. (2012)

^o^Schneider et al. (2012)

^p^Sugiyama (1969)

^q^Tomasello et al. (1985, 1989, 1994)

^r^van Hooff (1973)

Bolded form categories indicate the higher-order categories. The forms that are indented were summed with the higher-order categories in later analyses

**Table 7:** Adult and infant chimpanzees use the gesture Touch in significantly different contexts

Context	Infant initiators		Adult initiators	
	Frequency (%)	Adj. residual	Frequency (%)	Adj. residual
Attention getting**	14 (5%)	**3.0**	3 (1%)	**− 3.0**
Comfort*	10 (4%)	**2.5**	2 (1%)	**− 2.5**
Contact	35 (13%)	0.2	38 (12%)	− 0.2
Dominance/submission	2 (1%)	− 1.9	9 (3%)	1.9
Food beg/share/steal*	17 (6%)	**2.2**	8 (3%)	**− 2.2**
Greet**	16 (6%)	**− 3.1**	42 (14%)	**3.1**
Groom	15 (5%)	− 0.3	19 (6%)	0.3
Locomotion	2 (1%)	− 1.5	7 (2%)	1.5
Nurse***	22 (8%)	**5.1**	0 (0%	**− 5.1**
Play	117 (43%)	− 0.6	140 (45%)	0.6
Retrieve/Safeguard***	1 (< 0.5%)	**− 4.2**	22 (7%)	**4.2**
Other affiliative**	17 (6%)	**2.6**	6 (2%)	**− 2.6**
Undefined	5 (2%)	− 1.5	12 (4%)	1.5
Total	272 (100%)		308 (100%)	

Asterisk indicates context categories in which infant and adults differed significantly, with significant adjusted residuals in bold. * *p* < 0.05; ** *p* < 0.01; *** *p* < 0.001

Infant and adult chimpanzees used the gesture Touch in significantly different contexts, Chi-square (12) = 84.75, *p* < .001 (Table 7). Infants were significantly more likely than expected to use Touch in the contexts of Attention getting, Comfort, Food, and Nurse. Adults were significantly more likely than expected to use Touch in the contexts of Greet and Retrieve/Safeguard.

### Form by context

There were too many cells (82%) with low expected frequencies to conduct a statistical analysis of ten categories of form and 13 categories of context (Table S2). But we present here descriptive data on the presence or absence of forms within each context. Five different forms were found in Attention getting, seven in Comfort, eight in Contact, five in Dominance/submission, seven in Food, six in Greet, five in Groom, five in Nurse, all ten were found in Play, five in Retrieve/Safeguard, seven in Other affiliative, and three in Locomotion. In other words, an average of six different forms were found in the different contexts (range 3–10), and all contexts contained touches with different forms.

For the purposes of detecting statistical patterns, the contexts were further reduced to Play, Affiliative non-play (which include all affiliative contexts except play), and Non-Affiliative (which included undefined, Locomotion, Dominance/submission, and Food). In these analyses, the form of Feet was subsumed into Other. Forms of Touch varied by context, even when context was reduced to these three categories (Table 8), Chi-square = 73.18, *p* < .001. In the Play context, the form that occurred significantly more than expected was Hold with hand. In the Affiliative non-play context, Finger and Knuckles were the forms that occurred significantly more frequently than expected. In the Non-affiliative context, Finger tips occurred significantly more than expected.

**Table 8:** Forms of the gesture Touch differed significantly by context, reduced to three categories

Form	Context					
	Play		Affiliative non-play		Non-affiliative	
	Frequency	Adj. residual	Frequency	Adj. residual	Frequency	Adj. residual
Index finger	17	1.8	10	− 0.7	1	− 1.6
Finger*	32	**− 2.6**	60	**4.1**	7	**− 2.1**
Finger tips*	19	**− 2.0**	26	0.3	14	**2.4**
Knuckles**	4	**− 4.2**	26	**3.6**	7	1.0
Hand	109	0.6	96	− 0.7	33	0.2
Hand action*	6	− 0.1	3	− 1.6	5	**2.4**
Hold with hand**	52	**4.7**	15	**− 4.2**	8	− 0.8
Hold other	6	1.8	2	− 1.0	0	1.1
Other	12	0.8	7	− 1.2	4	0.5

Asterisk indicates form categories that differed significantly across contexts, * p < 0.05, ** p < 0.01, with significant adjusted residuals in bold

### Target location and context

There were too many cells (85%) with low expected frequencies to conduct a statistical analysis of 13 categories of target location and 13 categories of context. But the presence or absence of locations within each context is informative (Table S4). Six different locations were found in Attention getting, five in Comfort, 13 in Contact, seven in Dominance/submission, nine in Food, 13 in Greet, 12 in Groom, eight in Nurse, all 13 were found in Play, ten in Retrieve/Safeguard, seven in Other affiliative, and five in Locomotion. In other words, an average of nine different locations were found in the different contexts (range 5–13), and all contexts contained touches with different locations.

For the purposes of detecting statistical patterns, the contexts were further reduced to Play, Affiliative non-play, and Non-Affiliative (see above). Target location was significantly different across contexts, when reduced to these three contexts, Chi-square (24) = 74.52, *p* < .001 (Table 9). In the Play context, Touch was directed to the Foot, Leg, and Hand significantly more than expected. In the Affiliative non-play contexts, the target locations of Anogenital region and Side were significantly more frequent than expected. In the non-Affiliative contexts, Back was significantly more frequently the target location than expected.

**Table 9:** Target locations of the gesture Touch differed significantly by context (reduced to three categories)

Target location	Play		Affiliative non-play		Non-affiliative	
	Frequency	Adj. residual	Frequency	Adj. residual	Frequency	Adj. residual
Arm	27	− 1.7	37	1.1	13	0.9
Back*	25	− 1.9	33	0.5	16	**2.2**
Belly	12	− 0.6	12	− 0.4	7	1.5
Anogenital**	6	**− 2.9**	24	**4.1**	1	− 1.7
Chest	7	0.0	8	0.6	1	− 0.9
Chin	8	0.5	4	− 1.4	4	1.3
Face	13	− 0.9	14	− 0.3	8	1.6
Foot**	36	**3.3**	15	− **2.4**	4	− 1.4
Hand**	46	**2.2**	22	**− 3.1**	15	1.3
Head	26	1.3	20	− 0.2	3	− 1.6
Leg**	37	**2.7**	22	− 1.0	2	**− 2.5**
Neck	5	− 0.4	7	0.9	1	− 0.6
Side**	9	**− 2.9**	27	**3.4**	4	− 0.7

Asterisk indicates target location categories that differed significantly across contexts, * p < 0.05, ** p < 0.01, with significant adjusted residuals in bold

### Form–location patterns

We were interested in whether there was consistency in patterns of association between form and target location that might become evident by considering each context separately or considering each initiator separately. For the following two sets of analyses, descriptive data are presented for matrices that document different forms associated with different target location, which we call form–location patterns.

#### Within contexts

Returning to the consideration of 13 contexts, only those with at least 25 Touches were included in this consideration of form–location patterns. First, we considered the matrix composed of frequency of forms with the frequency of target locations within each context. For all of these considerations, Chi-square analyses were not permissible, due to the large number of cells with expected frequencies less than 5. But presenting the frequencies and percentages are informative in the assessment of flexibility in the form–location patterns of the gesture Touch.

In the Contact context, the chimpanzees used eight different forms and 13 different target locations (Table S3). The Hand (38%), Finger tips (18%), and Fingers (12%) were the predominant forms of the gesture Touch used in the Contact context, and Back (19%), Arm (18%), Hand (12%), and Leg (11%) were the primary target locations. In the form by target location matrix (104 cells), there were 38 different combinations with frequencies ranging from 1 to 6. The three cells with the highest frequencies (*n* = 6, 5, 5, respectively) were Hand touching Side, Hand touching Leg, and Knuckles touching Back.

In the Food context, the chimpanzees used seven different forms and nine different target locations (Table S5). The primary forms of the gesture Touch were Hand (40%) and Hold with the hand (24%). The primary target location was the Hand (28%), but five other body parts were targeted more than once. In the form by location matrix (63 cells), there were 17 different combinations, with the most prevalent being the form of Hold with the Hand and the target location of Hand (*n* = 4).

In the Greet context, the chimpanzees used six different forms and 13 different target locations of the gesture Touch (Table S6). The primary forms were Hand (48%), Finger (22%), and Knuckles (15%) and the primary target locations were Head (15%), Feet (12%), Anogenital region (10%), and Belly (10%). In the form by location matrix (78 cells), there were 30 different combination patterns found. The three cells with the highest frequencies were Hand touching the Feet of the social partner (*n* = 7); Hand touching the Head of the social partner (*n* = 5); and Finger touching the Head of the social partner (*n* = 4). Half of the remaining cells had only a single entry.

In the Groom context, the chimpanzees used five different forms and 12 different target locations (Table S7). The primary form of Touch was Finger (68%), with some Hand (15%). There were three primary target locations of the gesture Touch in the grooming context: Anogenital region (23%), Back (15%), and Arm (15%). In the form by location matrix (72 cells), there was one cell with five entries, Finger touching the Anogenital region (target location), one cell with four entries, Finger touching the Back, and 17 additional combinations (with frequencies of 3, 2, or 1).

In the Play context, the chimpanzees used the gesture Touch with all ten different forms and directed to all 13 different target locations (Table S8). The primary forms of Touch, in the context of Play, included Hand (42%), Hold with the Hand (20%), and Finger (13%). Touch was directed to the multiple target locations of Hand (18%), Feet (14%), Leg (14%), and Arm (11%). In the form by location matrix (130 cells), there were 62 different combinations, but none accounted for more than 8% of the Touches (*n* < 20). The three most frequently occurring form–location patterns were Hand touching Leg (*n* = 19), Hold with hand to the Hand of the social partner (*n* = 18), and Hand touching Back (*n* = 17).

When we reduced the number of contexts to three (Play, Affiliative non-play, and Non-affiliative) we still did not find any consistent form–location patterns. For Affiliative non-play contexts, there were 64 different combinations observed (out of 130 possible), but none account for more than 7% of the Touches in these contexts (*n* < 16). For the Non-affiliative contexts considered together, there were 36 different combinations observed (out of 117 possible), but none accounted for more than 8% of the Touches (*n* < 7).

#### Across initiator

Previous analyses found differences between infant and adult initiators in form and target location, so we consider form–location patterns separately for Touch initiated by infants and Touch initiated by adults. For Infants, all ten forms and all 13 target locations were used for the 273 Touches (Table S9). The three highest frequency cells were Hand touching the Arm of the social partner (*n* = 20), Hand touching Leg (*n* = 18), and Hand touching the Back of the social partner (*n* = 17). For Adults, there were 308 Touches, and nine forms were used (no Hold with other) and all 13 target locations were used (Table S10). For Adults, four cells had the highest frequencies, but these occurred between 15 and 17 times (< 5.5%): Hand touching Head, Hold with hand touching Leg, Hand touching Leg, and Hold with hand touching Hand of the social partner.

## Discussion

There was a great deal of flexibility and diversity found in the form and the use of the gesture Touch by the chimpanzees of this study. The chimpanzees displayed 36 different forms of the gesture, Touched 70 different target locations on the body of the social partner, and used the Touch gesture in 26 different contexts. Infants (2–5 years of age) differed significantly from the adults in form, location, and context. We did not find any consistent form–location patterns in any context, or when considering only Touches initiated by adults to infants, or only Touches initiated by infants. Therefore, we conclude that chimpanzees of this study showed flexibility and diversity in the use of the gesture Touch.

In line with the most widely used definition, we conclude Touch was flexible because it occurred in a wide variety of contexts. We found Touch occurred in 20 different contexts and six different blended contexts (Table 6). The focal subjects in our observations were young chimpanzees, so perhaps it is not surprising that we did not see Touch used in any aggressive contexts, any sexual contexts, and very few were used in Dominance/submission contexts. In order to be classified as flexible, it is a criterion that a gesture be used in multiple contexts, it is not necessary that the gesture be used in every context (e.g. Tomasello et al. 1985; Hobaiter and Byrne 2011). A predominance of contexts relating to affiliation, and not agonism would lend support to the idea that gestures are used flexibly since they convey non-urgent information (Tomasello and Zuberbuehler 2002). Future studies could determine whether the gestures conveying more ‘vital’ information are used more rigidly then those gestures conveying non-vital information.

A few previous studies concluded that Touch may be rigid in use, since Touch was only used in the nursing context, although there were also other gestures used to initiate nursing (e.g. Tomasello et al. 1985, 1989, 1994). Although we did find that only infants used the gesture Touch in the Nursing context (Table 7), we found that infant chimpanzees directed Touch to eight different locations on their mothers bodies, and used five different forms of Touch in the Nursing context. This is in contrast to the limited forms and/or limited target locations found for Touch in other studies (Tomasello et al. 1985, 1989, 1994; Liebal et al. 2004a). Therefore, we can conclude that, in this study, Touch was flexible in form, even in the Nursing context.

We found significant differences between the adults and the infants in the forms, target locations, and contexts of the gesture Touch. In consideration of 36 forms (Table 2), there were six forms not observed in infants, but 14 forms not observed in adults. All 18 target locations, however, were used by both infants and adults (Table 6). In consideration of 26 contexts, infants did not use Touch in five, and adults did not use Touch in six, notably, infants did not use the gesture Touch in the Safeguarding context and adults did not display Touch in the Nursing context. It is perhaps more interesting to note that where significant differences were found between infant’s and adult’s Touch, it was in the relative frequency of form, location, and context.

There was no apparent systematic change in how Touch was used from infancy to adulthood. In form, there was not a change to higher or lower specificity, as infants had a higher than expected use both of the hand and of the index finger, for example. In target location, there did not appear to be a systematic change, such as from less to more risky locations. Infants had higher than expected frequencies of Touches both to the back (less risky) and to the chin (very risky). In terms of context, it is notable that almost all of the contexts in which Touch occurred were affiliative, i.e. 22 of the 26 listed in Table 3, and Touch was not observed in any aggressive or sexual contexts in this study. Likely this is because (1) more affiliation than agonism is found in captive compared to wild settings (e.g. de Waal 1982); and (2) the focal subjects were young (between 2 and 5 years of age during the observations) when chimpanzees are rarely the recipients of aggression (e.g. Goodall 1986), and the gestures of adults were only recorded when they were interacting with these young infants. Finally, in contexts where Touch was used frequently, greater precision or specificity was not found in adult use compared to infant use, that is, approximately half of all possible form–target locations patterns were found in infant initiators and in adult initiators. Therefore, we conclude that there was not any systematic developmental change in the form or use of the gesture Touch.

Young chimpanzees in almost all settings spend a large amount of time engaged in social play with peers and the adults. Previous studies have identified play as the context with the most variety in gestures. It is therefore not surprising that a large percentage of Touch gestures were exhibited during play in the infants (43%), but it was surprising that the adults showed 45% of their Touches also in the context of play with infants (Table 7). It has long been proposed that social play is important, in part for young chimpanzees to learn the social rules required for proper adult socialization and understanding of the dominance hierarchy (Goodall 1986), but this suggests that adults are playing an active role as well.

Even in chimpanzees, there are cultural differences in the form, location, and/or use of some gestures (de Waal 1982; Goodall 1986; Whiten et al. 1999). By documenting the form and location of the gesture in this sample, we could then compare with those from other samples to investigate the details of cultural differences in gesture. Here we present the less well-known gesture, Touch, but since it has so much flexibility in form, it might be an especially useful foundation upon which cultural differences in other gestures could also emerge (Bard et al. 2005; Nakamura and Sakai 2014; Whiten et al. 1999).

This study could be interpreted as an attempt to determine the communicative meaning of the gesture Touch. It was possible to find that particular form–target locations communicated meaning in a more specific way than categorizing all forms into the general Touch. For example, when a chimpanzee uses a cupped hand, held palm up occasionally gently touching the mouth of another who is eating meat, the communicative message is clearly about food sharing, whereas the same form–target location in the context of a dominant’s approach can communicate submission or greeting. We found, instead, that for the gesture Touch, there are many form–location combinations within each context, that is, there were no particular combination patterns that were specific to any single context. Although we know that chimpanzees can produce gestures with specific and even symbolic meaning, we conclude that, in our study, chimpanzees did not use Touch to convey a single, specific communicative meaning.

It could be the case that Touch communicates to a particular social partner, a desire for attention to the self, and that chimpanzees then use another gesture or behaviour to communicate more specific information, sort of like calling out someone’s name in a crowded room and then waving them over. For example, Touch could draw attention from a potential playmate to the signaller, who then displays a play face, communicating a desire to play. Chimpanzees do use Gentle touch in gestural sequences, but it was found to be used as the first gesture as often as it was the second gesture, and it was much more frequently observed as a single gesture (Liebal et al. 2004a). A Touch, like a point, may have no intrinsic meaning in itself (e.g. Leavens et al. 2009; Tomasello et al. 2007), but be used in meaningful communication because the meaning is determined by psychological understanding of common ground (or joint attentional frame), a process that may be shared by humans and chimpanzees (Bard et al. 2014a; Bohn et al. 2016; Leavens et al. 2015). It may be necessary to document whether satisfactory outcomes occur to ascertain the communicative intention of a gesture (Cartmill and Byrne 2010; Genty et al. 2009; Graham et al. 2016; Hobaiter and Byrne 2014), and this consequence of Touch gesture was missing from our analyses here. Thus, these interpretations cannot be supported or ruled out for the gesture Touch, given the data analysed in our study, but they remain intriguing hypotheses.

It is possible that different form–target locations combinations might hold different meanings, which could be evident if the combinations were found differentially in different contexts. For instance, a hand, held palm up, touching the chin of a social partner could be a food sharing request if it occurred primarily in only the food context (but note that in our scheme, this gesture would not have been coded as Touch, but as Chin cup). We did not find any links between specific forms and specific contexts, between specific target locations and specific contexts, or between specific form–location combinations and specific contexts, which suggests that if there are associations of meaning with specific aspects of Touch, they are not linked globally with context.

It is difficult to conceive of a way to test which ontogenetic process best explains gestures based on our study of a single gesture, but we can exclude some processes as accounting for this particular gesture. If the form and context patterns were consistent from infancy to adulthood, or there were just a few different forms or a few form–location patterns then this study could have found supportive evidence for an innate channelling of the structure of the gesture Touch. A systematic change between use by adults and use by infants could be explained by the process of ontogenetic ritualization due to the uni-directional learning, e.g. greater specificity in adults, or a narrower range of form–location patterns. However, the wide range of forms of Touch, targeted to a wide range of locations, and without any systematic differences in use across contexts or any systematic change from infancy to adulthood, argues against both innate channelling and ritualization processes to explain the gesture Touch. In contrast to other tactile gestures, such as push or chin cup, we note that Touch is not a typical candidate for iconicity (Perlman et al. 2012; Savage-Rumbaugh et al. 1977; Tanner et al. 2006). Based on our findings, we can conclude that the gesture Touch, as a gesture type, is not iconic in the sense of mapping form to real world activities (e.g., Perlman and Gibbs 2013; Russon and Andrews 2011). We do not rule out the possibility, however, that some forms of Touch, alone or in combination with other gesture types, can be iconic, for example, to indicate desired goals of grooming or food sharing (Bard et al. 2014b; Goodall 1968; Pika and Mitani 2006; Plooij 1978).

This study is based on a relatively small sample size (3 young chimpanzees and 11 adults), but with long periods of observation (over 5 years). The age composition of the group was not representative of wild chimpanzee groups, although the total number of chimpanzees in the group was matched with that in the group at Bossou (Matsuzawa 2003, 2007). Some members of the KUPRI group have regular and direct contact with humans, which can, in principle, affect the variety and type of gestures produced (Leavens and Hopkins 1998; Leavens et al. 1996, 2004a, b, 2005; Roberts et al. 2014). We acknowledge that our findings might not generalize to other chimpanzee groups, but the variation in phenotypic outcomes due to exposure to different ecological and social environments is a general characteristic of plasticity found in both chimpanzees and humans (see for example, Bard and Leavens 2014; Boesch 2012).

## Conclusion

The results of this study show the extent of versatility in the use of the gesture Touch. Previous studies have found that gestures can be used flexibly (more than one gesture in each context, and each gesture is used in more than one context), with the meaning of a gesture based on the context in which it is used. This study, with a bottom-up design, is the first to demonstrate the extent of flexibility in the form, as well as context in which Touch occurs. The gesture Touch was used communicatively in Dominance/submission contexts, as well in as various types of affiliative contexts, to gain the attention of a social partner, to request food, to provide comfort, to initiate contact, and, of course, to play. Thus, if there is meaning in this gesture, it cannot be ascertained by knowledge only of the context, or only of the form. The results from this study illustrate the importance of contextualized meaning in understanding gesture use, and gesture flexibility of great apes. Perhaps communicative meaning of the gesture Touch is based on the prior establishment of common ground, but, without knowing outcomes, we cannot link gesture types with specific communicative intent. With a gesture being used so flexibly, not bound by form or context, it becomes imperative to understand the bigger picture to accurately access the communicative meaning conveyed by gestures in great apes.

## Electronic supplementary material

Below is the link to the electronic supplementary material.

Supplementary material 1 (DOCX 32 kb)
