# Supplementary material for: The gesture ‘Touch’: Does meaning-making develop in chimpanzees’ use of a very flexible gesture?
Source: Anim Cogn. 2017 Oct 24;22(4):535–50. doi: 10.1007/s10071-017-1136-0 (PMC6647888; doi:10.1007/s10071-017-1136-0)
Supplement: Supplementary file 1 — Supplementary material 1 (DOCX 32 kb) [file 10071_2017_1136_MOESM1_ESM.docx]

| Arm around back | Sits on top of | Hug | Grabs at | Somersault | Arm over head |
| --- | --- | --- | --- | --- | --- |
| Slaps at | Kick | Slap | Lip touch | Wave | Extend Arm |
| Kiss | Stomp ground | Presents | Raise arm | Shakes rope | Touch |
| Lies on top of | Hand on back | Rump present | Splash/throw water | Point | Tap |
| Reach back | Reach back with foot | Wrist present | Poke | Sway | Throw stuff |
| Tickle | Crouch | Chin cup | Slaps ground/wall | Bite | Grab |

Table S1: Touch is only one of the 36 gestures identified by Herring (2013)

Table S2: The form and context (10 by 13) matrix for all Touch gestures.

| **Context_new * form_new Crosstabulation** | | | | | | | | | | | | |
| --- | --- | --- | --- | --- | --- | --- | --- | --- | --- | --- | --- | --- |
| Count | | | | | | | | | | | | |
|  | | form_new | | | | | | | | | | Total |
|  |  | knuckles | finger tips | finger | hand | hand act | hold hand | hold other | feet | other | Index finger |  |
| Context_new | Att Get | 3 | 4 | 2 | 5 | 0 | 0 | 0 | 3 | 0 | 0 | 17 |
|  | Comfort | 3 | 2 | 1 | 2 | 1 | 2 | 0 | 1 | 0 | 0 | 12 |
|  | Contact | 8 | 13 | 9 | 28 | 0 | 8 | 2 | 1 | 0 | 4 | 73 |
|  | DomSub | 0 | 2 | 3 | 1 | 3 | 2 | 0 | 0 | 0 | 0 | 11 |
|  | Food | 2 | 4 | 1 | 10 | 0 | 6 | 0 | 1 | 0 | 1 | 25 |
|  | Greet | 9 | 4 | 13 | 28 | 0 | 3 | 0 | 0 | 1 | 0 | 58 |
|  | Groom | 0 | 1 | 23 | 5 | 0 | 0 | 0 | 0 | 3 | 2 | 34 |
|  | Nurse | 2 | 0 | 2 | 16 | 1 | 0 | 0 | 0 | 0 | 1 | 22 |
|  | Play | 4 | 19 | 32 | 109 | 6 | 52 | 6 | 7 | 5 | 17 | 257 |
|  | Retrieve | 2 | 3 | 7 | 10 | 1 | 0 | 0 | 0 | 0 | 0 | 23 |
|  | Other aff | 2 | 3 | 5 | 7 | 0 | 2 | 0 | 1 | 0 | 3 | 23 |
|  | undefined | 1 | 2 | 1 | 11 | 2 | 0 | 0 | 0 | 0 | 0 | 17 |
|  | Locomotion | 1 | 2 | 0 | 6 | 0 | 0 | 0 | 0 | 0 | 0 | 9 |
| Total | | 37 | 59 | 99 | 238 | 14 | 75 | 8 | 14 | 9 | 28 | 581 |

Table S3: The full target location by context (13 by 13) matrix for all Touch gestures

Target location TOTAL

Head Face Neck Chin Arm hand belly chest back side leg feet anogen

Context

Att Get 0 1 0 0 4 5 0 1 5 0 0 0 1 17

Comfort 0 1 0 0 1 1 0 0 7 2 0 0 0 12

Contact 6 4 3 1 13 9 1 1 14 7 8 3 3 73

DomSub 1 1 1 0 2 2 0 0 3 1 0 0 0 11

Food 0 3 0 4 4 7 1 0 2 1 2 1 0 25

Greet 9 4 1 2 4 5 6 4 3 4 3 7 6 58

Groom 2 3 1 0 5 2 1 1 5 2 2 2 8 34

Nurse 0 0 0 1 8 1 0 2 1 3 4 2 0 22

Play 26 13 5 8 27 46 12 7 25 9 37 36 6 257

Retrieve 3 2 2 0 4 2 2 0 1 5 0 1 1 23

Other aff 0 0 0 0 2 2 2 0 2 4 5 0 6 23

undefined 1 3 0 0 3 1 4 0 3 0 0 2 0 17

Locomotion 1 0 0 0 0 0 2 0 3 2 0 1 0 9

TOTAL 49 35 13 16 77 83 31 16 74 40 61 55 31 581

Table S4: The Form by Target Location matrix for the gesture Touch in the context of Contact

| **Location_new * form_new Crosstabulation** | | | | | | | | | | |
| --- | --- | --- | --- | --- | --- | --- | --- | --- | --- | --- |
| Count | | | | | | | | | | |
|  | | form_new | | | | | | | | Total |
|  |  | knuckles | finger tips | finger | hand | hold hand | hold other | feet | Index finger |  |
| Location_new | Head | 0 | 3 | 0 | 3 | 0 | 0 | 0 | 0 | 6 |
|  | Face | 1 | 1 | 1 | 1 | 0 | 0 | 0 | 0 | 4 |
|  | Neck | 0 | 0 | 0 | 3 | 0 | 0 | 0 | 0 | 3 |
|  | Chin | 0 | 1 | 0 | 0 | 0 | 0 | 0 | 0 | 1 |
|  | Arm | 1 | 1 | 1 | 4 | 4 | 0 | 0 | 2 | 13 |
|  | hand | 0 | 1 | 1 | 2 | 3 | 2 | 0 | 0 | 9 |
|  | belly | 0 | 1 | 0 | 0 | 0 | 0 | 0 | 0 | 1 |
|  | chest | 0 | 0 | 0 | 1 | 0 | 0 | 0 | 0 | 1 |
|  | back | 5 | 2 | 2 | 1 | 1 | 0 | 1 | 2 | 14 |
|  | side | 0 | 1 | 0 | 6 | 0 | 0 | 0 | 0 | 7 |
|  | leg | 1 | 1 | 1 | 5 | 0 | 0 | 0 | 0 | 8 |
|  | feet | 0 | 0 | 3 | 0 | 0 | 0 | 0 | 0 | 3 |
|  | anogen | 0 | 1 | 0 | 2 | 0 | 0 | 0 | 0 | 3 |
| Total | | 8 | 13 | 9 | 28 | 8 | 2 | 1 | 4 | 73 |

Table S5: The Form by Target location matrix for the gesture Touch in the context of Food

| **Location_new * form_new Crosstabulation** | | | | | | | | | |
| --- | --- | --- | --- | --- | --- | --- | --- | --- | --- |
| Count | | | | | | | | | |
|  | | form_new | | | | | | | Total |
|  |  | knuckles | finger tips | finger | hand | hold hand | feet | Index finger |  |
| Location_new | Face | 0 | 0 | 0 | 3 | 0 | 0 | 0 | 3 |
|  | Chin | 1 | 2 | 1 | 0 | 0 | 0 | 0 | 4 |
|  | Arm | 0 | 1 | 0 | 1 | 1 | 1 | 0 | 4 |
|  | hand | 0 | 0 | 0 | 2 | 4 | 0 | 1 | 7 |
|  | belly | 1 | 0 | 0 | 0 | 0 | 0 | 0 | 1 |
|  | back | 0 | 1 | 0 | 1 | 0 | 0 | 0 | 2 |
|  | side | 0 | 0 | 0 | 0 | 1 | 0 | 0 | 1 |
|  | leg | 0 | 0 | 0 | 2 | 0 | 0 | 0 | 2 |
|  | feet | 0 | 0 | 0 | 1 | 0 | 0 | 0 | 1 |
| Total | | 2 | 4 | 1 | 10 | 6 | 1 | 1 | 25 |

Table S6: The Form by Target Location matrix for the gesture Touch in the context of Greeting

| **Location_new * form_new Crosstabulation** | | | | | | | | |
| --- | --- | --- | --- | --- | --- | --- | --- | --- |
| Count | | | | | | | | |
|  | | form_new | | | | | | Total |
|  |  | knuckles | finger tips | finger | hand | hold hand | other |  |
| Location_new | Head | 0 | 0 | 4 | 5 | 0 | 0 | 9 |
|  | Face | 2 | 0 | 0 | 2 | 0 | 0 | 4 |
|  | Neck | 0 | 0 | 0 | 0 | 0 | 1 | 1 |
|  | Chin | 0 | 1 | 0 | 1 | 0 | 0 | 2 |
|  | Arm | 0 | 0 | 2 | 2 | 0 | 0 | 4 |
|  | hand | 1 | 1 | 0 | 1 | 2 | 0 | 5 |
|  | belly | 1 | 0 | 2 | 3 | 0 | 0 | 6 |
|  | chest | 1 | 2 | 0 | 1 | 0 | 0 | 4 |
|  | back | 2 | 0 | 0 | 1 | 0 | 0 | 3 |
|  | side | 2 | 0 | 1 | 1 | 0 | 0 | 4 |
|  | leg | 0 | 0 | 1 | 1 | 1 | 0 | 3 |
|  | feet | 0 | 0 | 0 | 7 | 0 | 0 | 7 |
|  | anogen | 0 | 0 | 3 | 3 | 0 | 0 | 6 |
| Total | | 9 | 4 | 13 | 28 | 3 | 1 | 58 |

Table S7: The Form by Target Location matrix for the gesture Touch in the context of Grooming

| **Location_new * form_new Crosstabulation** | | | | | | | |
| --- | --- | --- | --- | --- | --- | --- | --- |
| Count | | | | | | | |
|  | | form_new | | | | | Total |
|  |  | finger tips | finger | hand | other | Index finger |  |
| Location_new | Head | 0 | 2 | 0 | 0 | 0 | 2 |
|  | Face | 0 | 3 | 0 | 0 | 0 | 3 |
|  | Neck | 0 | 0 | 0 | 1 | 0 | 1 |
|  | Arm | 0 | 3 | 1 | 1 | 0 | 5 |
|  | hand | 0 | 2 | 0 | 0 | 0 | 2 |
|  | belly | 0 | 1 | 0 | 0 | 0 | 1 |
|  | chest | 0 | 1 | 0 | 0 | 0 | 1 |
|  | back | 0 | 4 | 0 | 0 | 1 | 5 |
|  | side | 1 | 0 | 1 | 0 | 0 | 2 |
|  | leg | 0 | 1 | 0 | 0 | 1 | 2 |
|  | feet | 0 | 1 | 1 | 0 | 0 | 2 |
|  | anogen | 0 | 5 | 2 | 1 | 0 | 8 |
| Total | | 1 | 23 | 5 | 3 | 2 | 34 |

Table S8: Form by target location matrix for the gesture Touch in the context of Play

| **Location_new * form_new Crosstabulation** | | | | | | | | | | | | |
| --- | --- | --- | --- | --- | --- | --- | --- | --- | --- | --- | --- | --- |
| Count | | | | | | | | | | | | |
|  | | form_new | | | | | | | | | | Total |
|  |  | knuckles | finger tips | finger | hand | hand act | hold hand | hold other | feet | other | Index finger |  |
| Location_new | Head | 0 | 2 | 2 | 12 | 2 | 1 | 0 | 1 | 0 | 6 | 26 |
|  | Face | 0 | 3 | 3 | 3 | 1 | 0 | 0 | 0 | 0 | 3 | 13 |
|  | Neck | 0 | 1 | 0 | 3 | 0 | 0 | 0 | 0 | 1 | 0 | 5 |
|  | Chin | 0 | 1 | 3 | 1 | 0 | 0 | 0 | 0 | 1 | 2 | 8 |
|  | Arm | 1 | 2 | 1 | 10 | 0 | 8 | 1 | 0 | 2 | 2 | 27 |
|  | hand | 1 | 2 | 6 | 14 | 1 | 18 | 3 | 0 | 0 | 1 | 46 |
|  | belly | 1 | 3 | 0 | 5 | 2 | 0 | 0 | 0 | 1 | 0 | 12 |
|  | chest | 0 | 1 | 0 | 3 | 0 | 0 | 0 | 0 | 0 | 3 | 7 |
|  | back | 0 | 0 | 2 | 17 | 0 | 0 | 1 | 5 | 0 | 0 | 25 |
|  | side | 1 | 3 | 2 | 3 | 0 | 0 | 0 | 0 | 0 | 0 | 9 |
|  | leg | 0 | 0 | 2 | 19 | 0 | 15 | 1 | 0 | 0 | 0 | 37 |
|  | feet | 0 | 1 | 11 | 13 | 0 | 10 | 0 | 1 | 0 | 0 | 36 |
|  | anogen | 0 | 0 | 0 | 6 | 0 | 0 | 0 | 0 | 0 | 0 | 6 |
| Total | | 4 | 19 | 32 | 109 | 6 | 52 | 6 | 7 | 5 | 17 | 257 |

Table S9: The Form by Target location matrix for the gesture Touch for Infant initiators

| **Location_new * form_new Crosstabulation** | | | | | | | | | | | | |
| --- | --- | --- | --- | --- | --- | --- | --- | --- | --- | --- | --- | --- |
| Count | | | | | | | | | | | | |
|  | | form_new | | | | | | | | | | Total |
|  |  | knuckles | finger tips | finger | hand | hand act | hold hand | hold other | feet | other | Index finger |  |
| Location_new | Head | 0 | 3 | 1 | 7 | 0 | 1 | 0 | 1 | 0 | 6 | 19 |
|  | Face | 0 | 3 | 5 | 6 | 3 | 0 | 0 | 0 | 0 | 3 | 20 |
|  | Neck | 0 | 0 | 1 | 6 | 0 | 0 | 0 | 0 | 2 | 0 | 9 |
|  | Chin | 1 | 2 | 5 | 1 | 0 | 0 | 0 | 0 | 1 | 2 | 12 |
|  | Arm | 2 | 1 | 6 | 20 | 1 | 3 | 1 | 3 | 0 | 3 | 40 |
|  | hand | 0 | 2 | 6 | 16 | 1 | 13 | 5 | 0 | 0 | 2 | 45 |
|  | belly | 1 | 2 | 0 | 2 | 1 | 0 | 0 | 0 | 1 | 0 | 7 |
|  | chest | 1 | 1 | 1 | 7 | 0 | 0 | 0 | 0 | 0 | 0 | 10 |
|  | back | 8 | 4 | 5 | 17 | 0 | 1 | 1 | 7 | 0 | 0 | 43 |
|  | side | 1 | 1 | 0 | 10 | 1 | 2 | 0 | 0 | 0 | 0 | 15 |
|  | leg | 0 | 0 | 1 | 18 | 0 | 3 | 1 | 0 | 0 | 1 | 24 |
|  | feet | 0 | 1 | 3 | 10 | 0 | 2 | 0 | 0 | 0 | 0 | 16 |
|  | anogen | 0 | 1 | 5 | 4 | 0 | 0 | 0 | 0 | 1 | 2 | 13 |
| Total | | 14 | 21 | 39 | 124 | 7 | 25 | 8 | 11 | 5 | 19 | 273 |

Table S10: The Form by Target location matrix for the gesture Touch for Adult initiators

| **Location_new * form_new Crosstabulation** | | | | | | | | | | | |
| --- | --- | --- | --- | --- | --- | --- | --- | --- | --- | --- | --- |
| Count | | | | | | | | | | | |
|  | | form_new | | | | | | | | | Total |
|  |  | knuckles | finger tips | finger | hand | hand act | hold hand | feet | other | Index finger |  |
| Location_new | Head | 0 | 3 | 9 | 15 | 3 | 0 | 0 | 0 | 0 | 30 |
|  | Face | 3 | 4 | 4 | 4 | 0 | 0 | 0 | 0 | 0 | 15 |
|  | Neck | 0 | 2 | 0 | 1 | 0 | 0 | 0 | 1 | 0 | 4 |
|  | Chin | 0 | 3 | 0 | 1 | 0 | 0 | 0 | 0 | 0 | 4 |
|  | Arm | 1 | 5 | 8 | 8 | 0 | 10 | 0 | 3 | 2 | 37 |
|  | hand | 3 | 4 | 5 | 9 | 0 | 17 | 0 | 0 | 0 | 38 |
|  | belly | 5 | 2 | 3 | 13 | 1 | 0 | 0 | 0 | 0 | 24 |
|  | chest | 0 | 2 | 0 | 1 | 0 | 0 | 0 | 0 | 3 | 6 |
|  | back | 7 | 3 | 3 | 11 | 3 | 0 | 1 | 0 | 3 | 31 |
|  | side | 3 | 5 | 6 | 10 | 0 | 0 | 0 | 0 | 1 | 25 |
|  | leg | 1 | 1 | 4 | 16 | 0 | 15 | 0 | 0 | 0 | 37 |
|  | feet | 0 | 3 | 13 | 14 | 0 | 8 | 1 | 0 | 0 | 39 |
|  | anogen | 0 | 1 | 5 | 11 | 0 | 0 | 1 | 0 | 0 | 18 |
| Total | | 23 | 38 | 60 | 114 | 7 | 50 | 3 | 4 | 9 | 308 |
